# Diagnostic value of baseline ^18^FDG PET/CT skeletal textural features in follicular lymphoma

**DOI:** 10.1038/s41598-021-03278-9

**Published:** 2021-12-10

**Authors:** Julie Faudemer, Nicolas Aide, Anne-Claire Gac, Ghandi Damaj, Jean-Pierre Vilque, Charline Lasnon

**Affiliations:** 1 Nuclear Medicine Department, Caen University Hospital, Caen, France; 2 UNICAEN, INSERM 1086 ANTICIPE, Normandy University, Caen, France; 3 Haematology Institute, Caen University Hospital, Caen, France; 4 Haematology Institute, Comprehensive Cancer Centre François Baclesse, UNICANCER, Caen, France; 5 Nuclear Medicine Department, Comprehensive Cancer Centre François Baclesse, UNICANCER, 3 Avenue du General Harris, BP 45026, 14076 Caen Cedex 5, France

**Keywords:** Cancer imaging, B-cell lymphoma, Tumour heterogeneity

## Abstract

At present, ^18^F-fluorodesoxyglucose (^18^FDG) positron emission tomography (PET)/computed tomography (CT) cannot be used to omit a bone marrow biopsy (BMB) among initial staging procedures in follicular lymphoma (FL). The additional diagnostic value of skeletal textural features on baseline ^18^FDG-PET/CT in diffuse large B-cell lymphoma (DLBCL) patients has given promising results. The aim of this study is to evaluate the value of ^18^FDG-PET/CT radiomics for the diagnosis of bone marrow involvement (BMI) in FL patients. This retrospective bicentric study enrolled newly diagnosed FL patients addressed for baseline ^18^FDG PET/CT. For visual assessment, examinations were considered positive in cases of obvious bone focal uptakes. For textural analysis, the skeleton volumes of interest (VOIs) were automatically extracted from segmented CT images and analysed using LifeX software. BMB and visual assessment were taken as the gold standard: BMB −/PET − patients were considered as bone-_NEGATIVE_ patients, whereas BMB +/PET −, BMB −/PET + and BMB +/PET + patients were considered bone-_POSITIVE_ patients. A LASSO regression algorithm was used to select features of interest and to build a prediction model. Sixty-six consecutive patients were included: 36 bone-_NEGATIVE_ (54.5%) and 30 bone-_POSITIVE_ (45.5%). The LASSO regression found variance__GLCM_, correlation__GLCM_, joint entropy__GLCM_ and busyness__NGLDM_ to have nonzero regression coefficients. Based on ROC analysis, a cut-off equal to − 0.190 was found to be optimal for the diagnosis of BMI using PET pred.score. The corresponding sensitivity, specificity, PPV and NPV values were equal to 70.0%, 83.3%, 77.8% and 76.9%, respectively. When comparing the ROC AUCs with using BMB alone, visual PET assessment or PET pred.score, a significant difference was found between BMB versus visual PET assessments (*p* = 0.010) but not between BMB and PET pred.score assessments (*p* = 0.097). Skeleton texture analysis is worth exploring to improve the performance of ^18^FDG-PET/CT for the diagnosis of BMI at baseline in FL patients.

## Introduction

Follicular lymphoma (FL) is the most common indolent B-cell lympho-proliferative disorder of transformed follicular centre B cells, accounting for 20–25% of adult non-Hodgkin’s lymphomas (HLs) worldwide^1^. Follicular lymphoma is characterized by diffuse lymphadenopathy, splenomegaly and often bone marrow involvement (BMI)^2^. Indeed, BMI defined by a positive bone marrow biopsy (BMB) has been reported in 52% to 55% of newly diagnosed FL patients^3–6^. BMI is an important factor in most clinical risk stratification indices, including the Follicular Lymphoma International Prognostic Index (FLIPI). The presence of BMI can change the treatment strategy, especially in patients who were thought to have early-stage disease prior to having a bone assessment. ^18^F-fluorodesoxyglucose (^18^FDG) positron emission tomography (PET)/computed tomography (CT) is now used for the assessment of BMI in HL patients^7,8^ and DLBCL patients^9–13^. Indeed, several studies have demonstrated sufficient diagnostic performances for these two histologic subtypes of lymphoma. In FL, data are sparse from smaller series of patients and display weaker diagnostic performances for BMI^13–17^. More specifically, the incidence of cases of positive BMB with negative visual PET examinations has been estimated to be 13%^18^. Therefore, PET is generally not used for BMI assessment in follicular lymphomas, and BMB arbitrarily taken from the iliac crest is preferred as the gold standard. However, the main shortcomings of BMB are inadequate sampling and possible pain, bleeding or infection complications^19^. Some studies have shown that a combination of PET and BMB could be an alternative for a more accurate BMI assessment than either PET or BMB alone^20,21^.

Today, there is a growing interest in haematology in using alternatives to visual or semiquantitative PET assessments that are based on textural features (TFs)^22,23^. Indeed, the basic visual interpretation of diffuse bone marrow involvement without focal bone lesions on PET can be difficult, leading to false-negative results. The diagnostic value of skeletal TFs compared to BMB and PET visual analysis on baseline ^18^FDG PET/CT in DLBCL patients has been demonstrated^24^. By extrapolation, we assume that the quantification of the metabolic heterogeneity of the skeleton could also significantly improve the bone pretherapeutic evaluation in FL patients. Therefore, the aim of this study was to evaluate the value of textural features (TFs) for the diagnosis of BMI.

## Results

### Population characteristics

**Figure 1:**
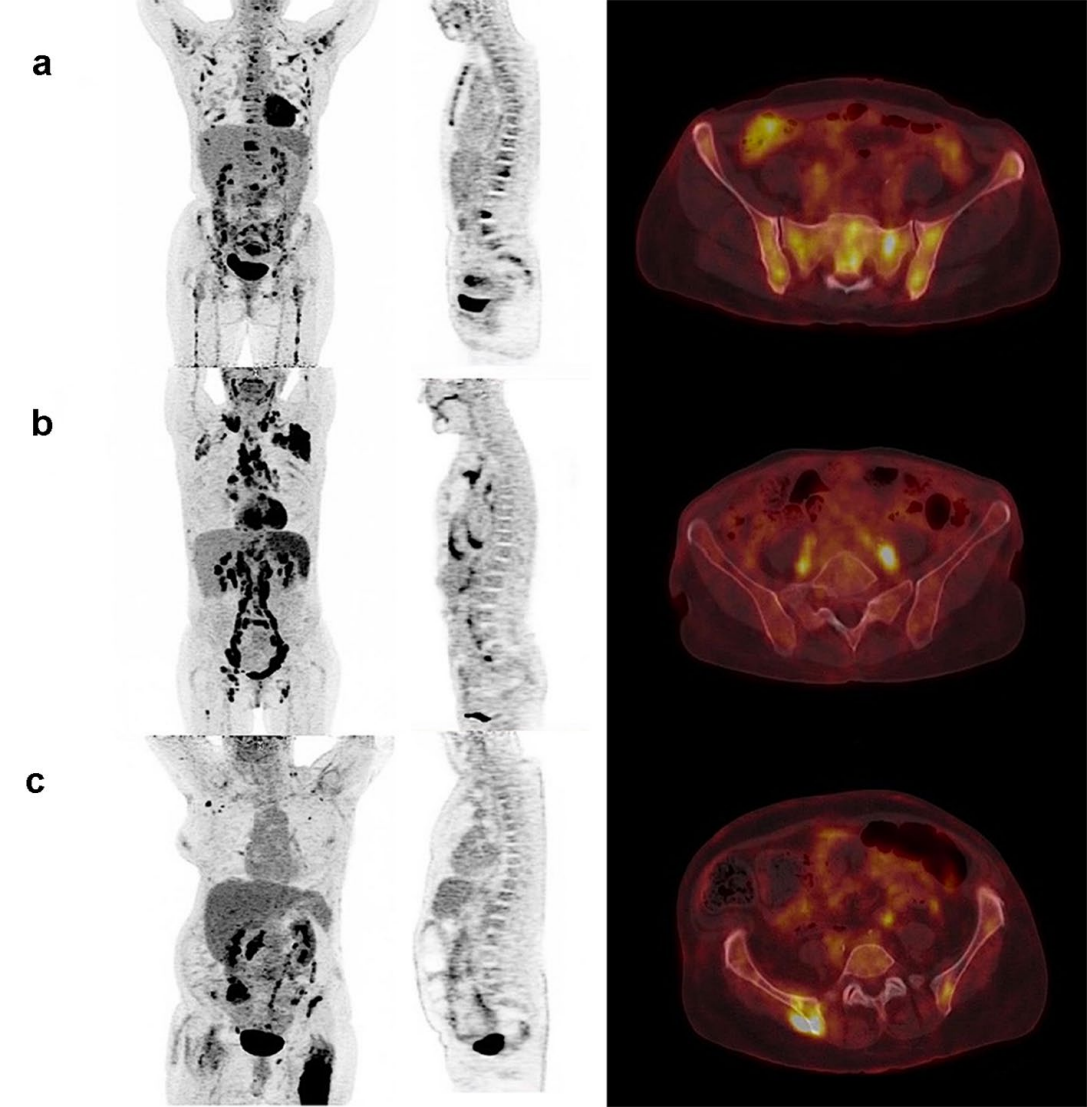
Representative MIP PET, sagittal PET and axial PET/CT images of patients PET +/BMB + (**a**); PET −/BMB + (**b**) and PET +/BMB − (**c**).

From the 113 FL patients identified from our database, 66 patients were ultimately included. Twenty-one patients were excluded because of missing BMB and 26 because of missing baseline ^18^FDG PET/CT. Fifty-nine patients were scanned on the Biograph TrueV Pet system, and seven were scanned on the Vereos PET system. There were 36 bone-_NEGATIVE_ patients (54.5%) and 30 bone-_POSITIVE_ patients (45.5%). Among the bone-_POSITIVE_ patients, there were four BMB −/PET_VISU_ + patients (13.3%), 14 BMB +/PET_VISU_− patients (46.7%) and 12 BMB +/PET_VISU_ + patients (40.0%). Focusing on BMB −/PET_VISU_ + patients, hypermetabolic lesions were located towards the axial skeleton and not in the appendicular skeleton, explaining the negativity of the BMB. Representative examples of each case are shown in Fig. 1.

The population characteristics are summarized in Table 1. Among the bone-_NEGATIVE_ patients, there were 4 patients (11.1%) staged 1, 10 (27.8%) staged 2, 13 (36.1%) staged 3 and 9 (25.0%) staged 4. There was no difference in the technical PET parameters between the bone-_NEGATIVE_ and bone-_POSITIVE_ groups of patients. The mean injected dose (MBq/kg), uptake time (min) and glycaemia (g/l) were 289 ± 54.5 versus 287 ± 56.3 (*p* = 0.61), 58.32 ± 3.59 versus 59.15 ± 3.52 (*p* = 0.30) and 1.06 ± 0.29 versus 0.98 ± 0.11 (*p* = 0.21), respectively. Hip prostheses were encountered in only four patients, two with a unilateral hip prosthesis and two with a bilateral hip prosthesis. No other types of prosthesis were encountered.

**Table 1:** Patients’ characteristics.

Statistic	n	All	n	Bone-_NEGATIVE_	n	Bone-_POSITIVE_	*p* value
Age, mean (SD)	66	59.9 (9.9)	36	60.2 (10.1)	30	59.6 (10.0)	0.959
Body Mass Index (kg/m^2^), mean (SD)	66	26.3 (4.7)	36	26.8 (5.2)	30	25.7 (4.0)	0.449
**Sex, n (%)**							
Women	66	32 (48.5)	36	16 (44.4)	30	16 (53.3)	0.621
Men		34 (51.5)		20 (55.6)		14 (46.7)	
**FLIPI score, n (%)**							
0–1	66	34 (51.5)	36	19 (52.8)	30	15 (50)	0.0004
2–3		23 (34.8)		16 (44.4)		7 (23.3)	
4–5		9 (13.6)		1 (2.8)		8 (26.7)	
**Bulky, n (%)**							
No (6 cm)	66	45 (68.2)	36	26 (72.2)	30	19 (63.3)	0.596
Yes (> 6 cm)		21 (31.8)		10 (27.8)		11 (36.7)	
**First-line treatment, n (%)**							
R-CHOP	66	40 (60.6)	36	19 (52.8)	30	21 (70.0)	0.031
Rituximab		16 (24.2)		12 (33.3)		4 (13.3)	
R-bendamustine		4 (6.1)		0 (0)		4 (13.3)	
Obinutuzumab-Lenalidomide		3 (4.5)		2 (5.6)		1 (3.3)	
R-ABVD		1 (1.5)		1 (2.8)		0 (0)	
Radiotherapy		1 (1.5)		1 (2.8)		0 (0)	
Follow-up		1 (1.5)		1 (2.8)		0 (0)	
Hb (g/dl), mean (SD)	61	12.8 (2.2)	33	13.3 (1.77)	28	12.3 (2.52)	0.127
WBC (G/l), mean (SD)	61	6.79 (3.08)	33	6.68 (2.59)	28	6.93 (3.65)	0.717
Platelets (G/l), mean (SD)	60	219.68 (111.68)	31	234.34 (103.87)	29	204.01 (121.14)	0.164
LDH (UI/l), mean (SD)	39	223.0 (90.7)	23	195.5 (42.5)	16	262.6 (126.2)	0.117
β2-microglobulin (mg/l), mean (SD)	17	3.34 (1.99)	10	2.42 (0.40)	7	4.68 (2.74)	0.006
Albumin (g/l), mean (SD)	41	39.53 (5.62)	23	40.56 (5.50)	20	38.34 (5.80)	0.164
Ca^2+^ (mmol/l), mean (SD)	43	2.32 (0.12)	24	2.34 (0.11)	19	2.29 (0.13)	0.111
Alkaline phosphatase (UI/l), mean (SD)	48	88.10 (52.77)	27	74.3 (24.20)	21	105.8 (73.06)	0.045

*n* number of observations, *BMI* body mass index, *Hb* haemoglobin, *WBC* white blood cells, *LDH* serum lactate dehydrogenase.

### Validation of previous results in the field

**Figure 2:**
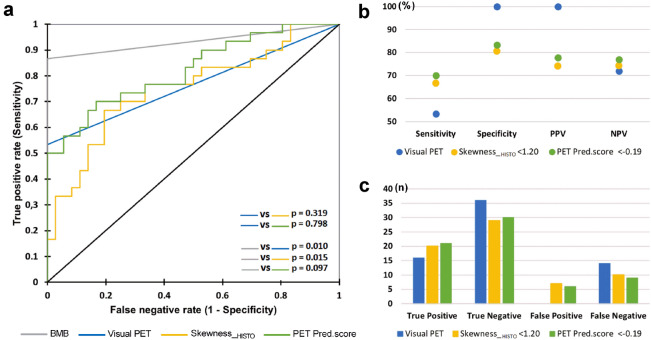
ROC curves for the diagnosis of BMI using BMB, visual PET, skewness__HISTO_ PET and pred.score PET assessments. (**a**) ROC curves comparison; (**b**) sensitivity, specificity, positive predictive value (PPV), and negative predictive values (NPV); (**c**) true positive, true negative, false positive and false negative rates.

A previous study^24^ found skewness__HISTO_ to be a promising PET parameter to discriminate between bone-_NEGATIVE_ and bone-_POSITIVE_ DLBCL patients with a cut-off value set to 1.26. In the present database of FL patients, the optimal cut-off for skewness__HISTO_ was 1.20 (AUC = 0.750 [− 95% CI = 0.629–0.871], *p* < 0.0001), with sensitivity, specificity, PPV, NPV and accuracy values of 66.7%, 80.6%, 74.1%, 74.4% and 74.2%, respectively (Fig. 2). Among the 10 false-negative results, 3 would have been reclassified as positive on a visual PET assessment basis because of lesions located out of the field of analysis, especially on the costal grill or vertebra atlas and axis. Seven false-positive results were also observed.

### Multivariable diagnostic value of PET radiomics for bone involvement at baseline staging

**Figure 3:**
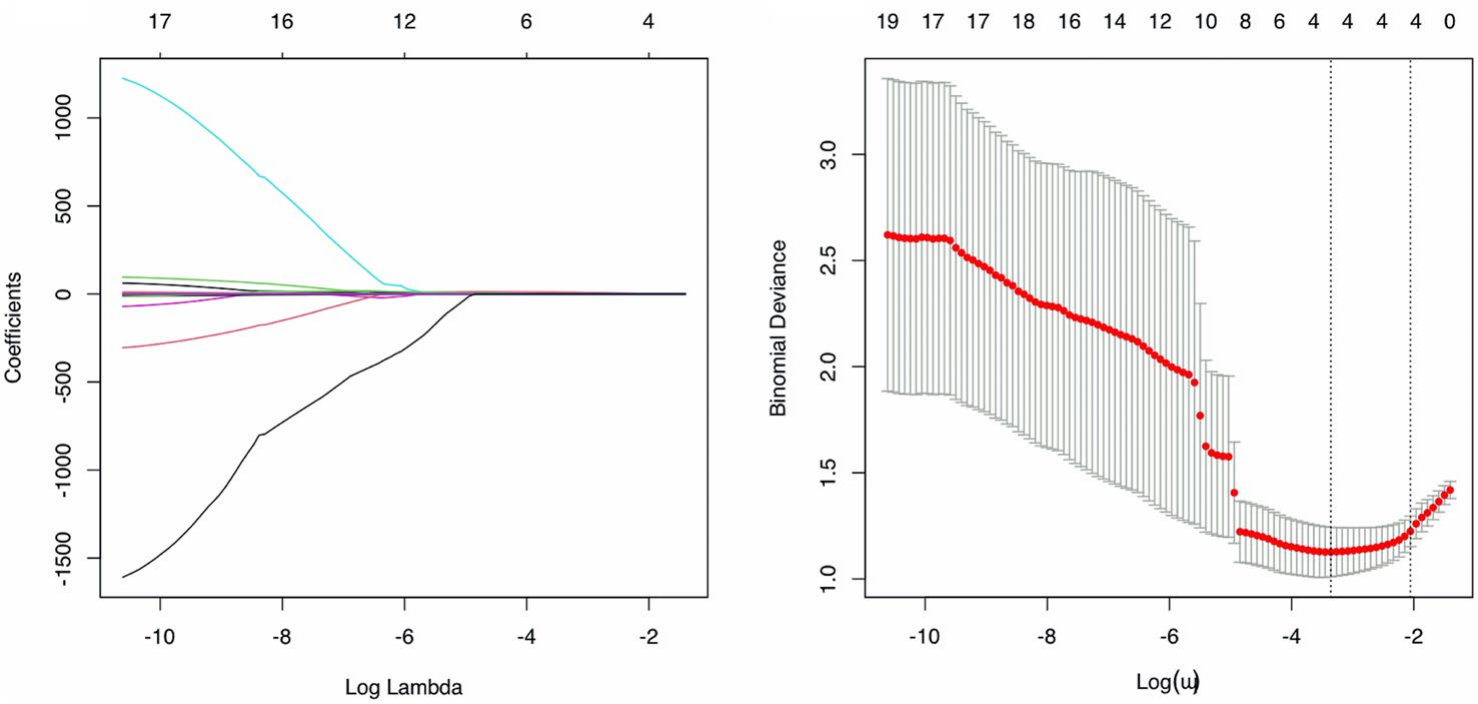
Coefficient (left panel) and cross-validation (right panel) plots of the LASSO analysis.

**Figure 4:**
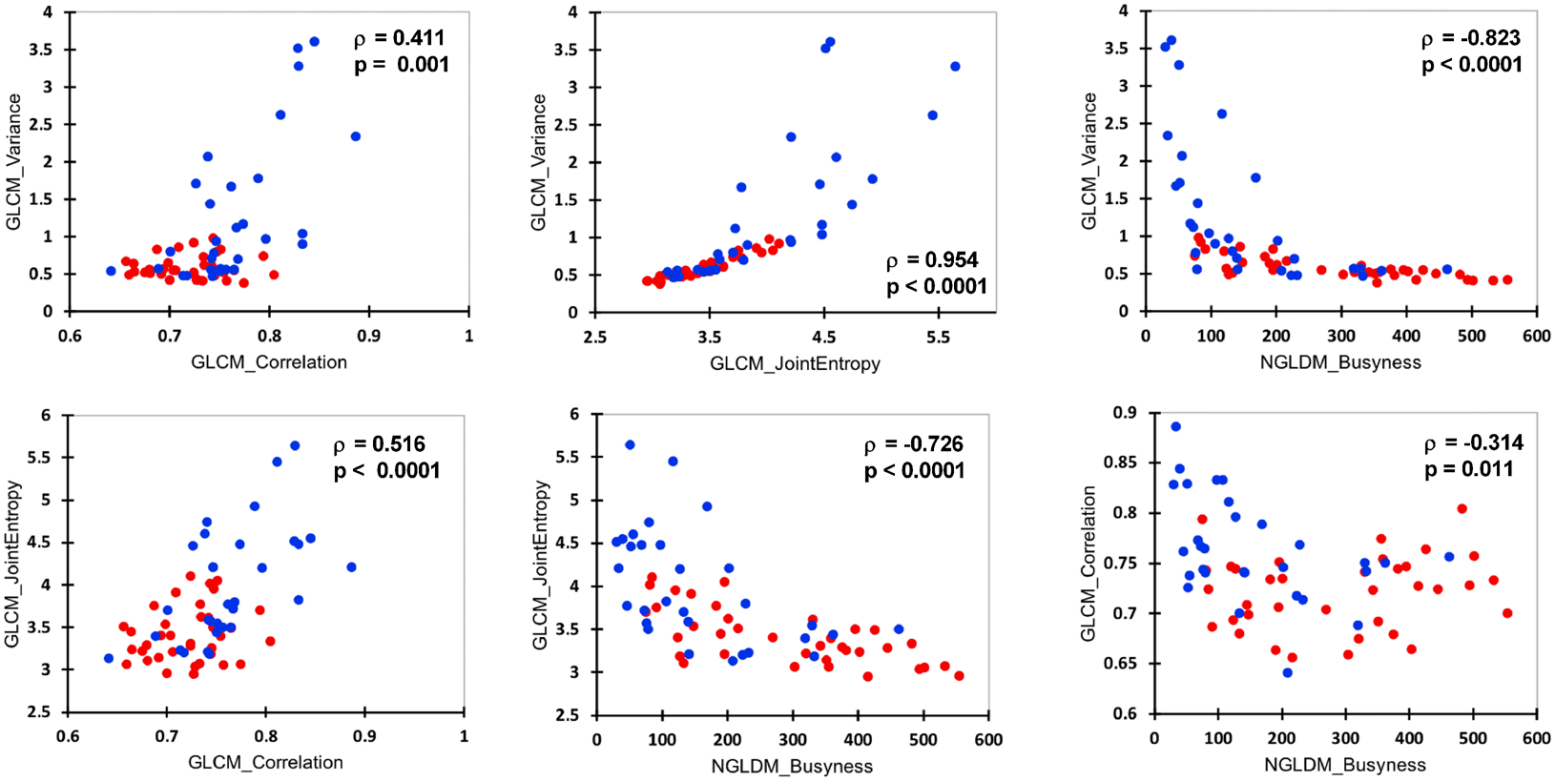
Correlation plots between PET radiomics retained by the LASSO analysis. Red dots represent bone-_NEGATIVE_ patients, and blue dots represent bone-_POSITIVE_ patients.

Thirteen ^18^FDG PET/CT variables out of the 26 analysed were significantly different between the bone-_NEGATIVE_ and bone-_POSITIVE_ patients (Table 2). The LASSO regression including all analysed PET radiomics (n = 26) found variance__GLCM_, correlation__GLCM_, joint entropy__GLCM_ and busyness__NGLDM_ to have nonzero regression coefficients. Coefficient and cross-validation plots are provided in Fig. 3. Correlations between these four PET radiomics scans can be seen in Fig. 4. The corresponding linear equation for the computation of the prediction score was as follows:

$$\begin{aligned} Pred.score & = - 8.134 + 0.927 \times variance_{GLCM} + 10.272 \times correlation_{GLCM} \\ & \quad + 0.076 \times joint entropy_{GLCM} - 0.003 \times busyness_{NGLDM} \\ \end{aligned}$$

**Table 2:** PET characteristics for the entire series, for bone-_NEGATIVE_ and for bone-_POSITIVE_ patients.

Statistic	All (n = 66)		Bone-_NEGATIVE_ (n = 36)		Bone-_POSITIVE_ (n = 30)		*p* value *
	Mean	SD	Mean	SD	Mean	SD	
**Conventional PET parameters**							
SUV_max_	9.312	13.080	5.639	3.035	13.720	18.478	**0.0003**
SUV_peak_	5.171	5.999	3.203	1.462	7.531	8.291	** < 0.0001**
SUV_Skewness_	2.236	4.806	1.141	0.535	3.549	6.997	**0.0004**
SUV_Kurtosis_	42.684	229.499	6.934	7.053	85.584	341.055	0.005
SUV_ExcessKurtosis_	39.684	229.499	3.934	7.053	82.584	341.055	0.005
TLG (mL)	4011.293	1202.959	3731.603	966.793	4346.921	1397.038	0.096
**GLCM PET parameters**							
Inverse Difference	0.763	0.045	0.780	0.031	0.742	0.052	**0.0019**
Angular Second Moment	0.131	0.040	0.146	0.033	0.113	0.040	**0.0016**
Variance	0.906	0.724	0.591	0.154	1.285	0.944	** < 0.0001**
Correlation	0.741	0.049	0.721	0.037	0.764	0.052	**0.0004**
Joint Entropy	3.678	0.583	3.416	0.325	3.993	0.676	**0.0001**
Dissimilarity	0.545	0.166	0.474	0.081	0.630	0.203	**0.0003**
**NGLDM PET parameters**							
Coarseness	0.00011	0.00003	0.00010	0.00003	0.00011	0.00004	0.353
Contrast	0.008	0.005	0.008	0.003	0.008	0.007	0.284
Busyness	226.322	146.045	286.996	145.528	153.513	113.701	** < 0.0001**
**GLZLM PET parameters**							
SZE	0.428	0.032	0.420	0.021	0.438	0.041	0.093
LZE	1,156,944.857	979,824.455	1,340,375.967	1,013,772.843	936,827.525	923,414.112	0.035
LGZE	0.236	0.092	0.260	0.094	0.207	0.085	0.022
HGZE	32.188	39.203	17.590	6.664	49.706	53.498	** < 0.0001**
SZLGE	0.098	0.036	0.109	0.035	0.086	0.034	0.012
SZHGE	18.002	29.692	8.106	4.141	29.876	41.452	** < 0.0001**
LZLGE	196,790.157	208,297.063	237,931.936	209,422.317	147,420.022	202,953.085	0.036
LZHGE	8,734,870.919	6,280,967.605	9,711,056.721	6,747,413.040	7,563,447.956	5,677,537.181	0.088
GLNU	221.652	48.709	233.464	51.065	207.477	43.259	0.059
ZLNU	300.057	193.919	235.765	74.066	377.207	260.141	0.008
ZP	0.015	0.007	0.013	0.002	0.018	0.010	**0.001**

*With Bonferroni correction a *p* value < 0.002 was considered statistically significant. Bold *p* values are significant.

The mean pred.score of the entire series was equal to − 0.096 ± 1.383. Based on ROC analysis, a cut-off equal to − 0.190 was found to be optimal for the diagnosis of BMI: AUC = 0.822 (95% CI = 0.721–0.924, *p* < 0.0001). The corresponding sensitivity, specificity, PPV and NPV values were equal to 70.0%, 83.3%, 77.8% and 76.9%, respectively (Fig. 2). Twenty-seven patients had a pred.score >  − 0.190 and were considered positive for BMI, among which six were false-positive results (BMB −/PET − patients). Additionally, nine false-negative results were observed, including seven BMB +/PET_VISU_ − patients and two BMB +/PET_VISU_ + patients whose lesions were out of the field of quantitative PET analysis. These patients could be easily recovered by visual analysis: one with a lesion on the costal grill and the other with a lesion on the upper jaw. *In fine*, only 7 bone-_POSITIVE_ patients (23.3%) would have been missed using skeletal PET quantification analysis. When comparing the AUCs from ROC analyses for BMI assessment with BMB alone, visual PET alone, PET skewness__HISTO_ alone and PET pred.score (Fig. 2, Table 3), significant differences were found between BMB and visual PET assessments (*p* = 0.010) and between BMB and PET skewness__HISTO_ assessments (*p* = 0.015). No difference was observed between BMB and PET pred.score assessments (*p* = 0.097). No difference was found among the PET pred.score, visual PET alone, or PET skewness__HISTO_ regarding the assessment of BMI.

**Table 3:** ROC curves results regarding the diagnosis of BMI using BMB, visual PET, skewness__HISTO_ PET and pred.score PET assessments.

	AUC	Standard error	Lower bound (95%)	Upper bound (95%)	*p* value
BMB	0.933	0.302	0.871	0.995	< 0.0001
Visual PET	0.767	0.046	0.676	0.857	< 0.0001
Skewness__HISTO_	0.750	0.062	0.629	0.871	< 0.0001
Pred.score PET	0.822	0.052	0.721	0.924	< 0.0001

*AUC* area under the curve, *BMB* bone marrow biopsy, *ROC* receiver operating characteristic, *BMI* bone marrow involvement, *PET* positron emission tomography.

The correlation between selected PET radiomics and biological characteristics was explored and is summarized in Table 4. Significant negative correlations were found between haemoglobin blood level and variance__GLCM_ (ρ = -0.447, *p* = 0.0003) and joint entropy__GLCM_ (ρ = -0.498, *p* < 0.0001).

**Table 4:** Correlations between biological variables and PET variables retained for pred.score computation.

	Variance__GCLM_		Correlation__GLCM_		JointEntropy__GLCM_		Busyness__NGLDM_	
	ρ coefficient	*p* value*	ρ coefficient	*p* value*	ρ coefficient	*p* value*	ρ coefficient	*p* value*
Haemoglobin (g/dl)	− **0.447**	**0.0003**	− 0.357	0.005	− **0.498**	** < 0.0001**	0.342	0.007
WBC (G/L)	0.109	0.400	0.096	0.460	0.093	0.474	− 0.182	0.161
Platelets (G/l)	0.112	0.395	− 0.115	0.382	0.028	0.832	− 0.187	0.152
LDH (UI/l)	0.383	0.017	0.245	0.133	0.306	0.059	− 0.281	0.083
ß2-microglobulin (mg/l)	0.421	0.094	0.090	0.733	0.442	0.077	− 0.274	0.287
Albumin (g/l)	− 0.372	0.015	− 0.223	0.150	− 0.412	0.006	0.316	0.040
Calcium (mmol/l)	− 0.444	0.003	− 0.272	0.078	− 0.424	0.005	0.391	0.010
Alkaline phosphatase (UI/l)	− 0.011	0.943	0.128	0.385	− 0.038	0.799	− 0.094	0.523

*With Bonferroni correction a *p* value < 0.001 was considered statistically significant. Bold *p* values are significant. LDH: serum lactate dehydrogenase; WBC: white blood cells.

## Discussion

The aim of the present study was to extrapolate previous results obtained for the diagnosis of BMI using PET radiomics in DLBCL patients to FL patients.

Skewness was previously found to be a promising parameter for the identification of patients with BMB involvement without visually assessable focal lesions, with a positive LR of 4.46. Interestingly, in our series of FL patients, the optimal cut-off value was consistent: equal to 1.20 versus 1.26 previously in DLBCL. However, skewness__HISTO_ BMI diagnostic performances were not as impressive, with low additional value over visual PET assessment alone: the sensitivity and NPV were 66.7% versus 53.3% and 74.4% versus 72.0% for skewness__HISTO_ and visual PET assessments, respectively. Well-known differences in metabolic characteristics between FL and DLBCL diseases could explain these results. In particular, FL uptake is usually less intense than that of DLBCL^25^. Another issue could be the important discrepancies in BMI at diagnosis between DLBCL and FL patients, with the rate of positive BMB estimated to be 15% in newly diagnosed DLBCL and 50% in FL^6^. Additionally, cases of BMB +/PET − patients were previously estimated to be only 3.1% in DLBCL^26^ but were estimated to be 13% in FL patients^18^. It is worth noting that this rate was even slightly superior in our series, reaching 21% of patients. However, it should be emphasized that cases of pure diffuse FDG uptake were considered positive in the study performed by Nakajima et al., whereas they were considered negative in the present study, which could partly explain this difference. Moreover, BMB −/PET + patients were also estimated at 13% and 12% in the another publication^26^, meaning that BMB could be tricked.

All things considered, it seemed to us that a multivariable approach using radiomics could be more accurate.

As has been highlighted in the literature, radiomic index values are highly dependent on the segmentation method^27–30^. The CT bone segmentation methods used here to draw VOIs were semiautomatic, with very little manual intervention and had already been shown to have a great interobserver agreement, which guaranteed their robustness^24^. To continue methodological considerations, the robustness of the radiomic indices to the intensity discretization method has been widely evaluated in the literature. Indices can be compared only if the same calculation parameters are used, which is the case here due to absolute resampling^31^.

In doing so, variance__GLCM_, correlation__GLCM_, joint entropy__GLCM_ and busyness__NGLDM_ were identified by LASSO analysis as potential variables of interest to build a linear model of prediction. None of the histogram or size-zone matrices were retained. It seemed that parameters extracted from GLCM or NGLDM were ideal candidates for describing skeletal tumour heterogeneity. Unlike histogram-based indices, calculated from original images, they reflect the spatial arrangement of voxel intensities. Even though statistical significance was not reached, with an optimal threshold PET pred.score set to − 0.19, the sensitivity and NPV were improved compared to visual PET assessment alone: 70.0% versus 53.3% and 76.9% versus 72.0%, respectively (Fig. 2). Finally, even if the performance of the BMB appeared to be better than that of the PET pred.score, it was still notable that there was no statistically significant difference between the ROC curves AUCs of these two diagnostic tests (*p* = 0.097). This may suggest that PET pred.score BMI assessment could perform equally to BMB provided that the model is strengthened with a larger database.

Notably, some examinations were found to be negative in terms of the PET pred.score but positive on visual PET assessment because of lesions located outside the VOIs. This result means either that improvement in CT bone segmentation has to be made to encompass the whole skeleton or that visual and quantitative PET assessments have to be conjointly made. The current paradigm of radiomic analysis adds quantitative information to visual analysis or biology without totally replacing them^32^, and it seems that the best option would be to combine visual and quantitative PET assessments. Presently, using this combined strategy, 7 bone__POSITIVE_ patients would have been missed compared to 14 patients using visual PET alone.

A more complex strategy combining clinical, biological and PET features should also be explored. However, the number of patients included in the present study did not allow us to test such strategies. We still looked for correlations between biological PET variables and found significant negative correlations between haemoglobin level and variance__GLCM_ and joint entropy__GLCM_. Some studies have demonstrated that marrow hypermetabolism correlates with leukocyte and neutrophil levels, both of which are associated with a poor response to treatment^33,34^, but this was not observed in our series.

Furthermore, the limited number of included patients did not allow the performance of the internal test. Therefore, the reliability of such a model should be evaluated on an independent dataset, ideally acquired on a different PET system or from a different centre, for its performances to be definitely validated.

Applying a multivariable PET radiomics model to baseline ^18^FDG PET/CT images could be a promising path to improve the diagnosis of BMI follicular lymphoma patients. Prospective and larger clinical studies are needed to strengthen the model and to definitively confirm this hypothesis.

## Methods

### Population

In this retrospective double-centre study, we enrolled 113 patients newly diagnosed with FL from November 2014 to May 2019 who were treated with a chemotherapy regimen. The inclusion criteria were as follows: patients over 18 years old, histopathologically proven FL, pretherapeutic bone marrow biopsy and ^18^FDG PET/CT. Clinical variables, including age at diagnosis, sex, body mass index, Ann Arbor stage, bulky mass, FLIPI score, first-line treatment type, serum haemoglobin level, serum platelet level, serum white cell level, serum β2-microglobulin (β2M) level, serum lactate dehydrogenase (LDH) level, serum albumin level, serum calcium level and serum alkaline phosphatase level, were recorded. All procedures performed in studies involving human participants were approved by the local ethics committee and were in accordance with the 1964 Helsinki Declaration. In accordance with European regulations, observational studies without any additional therapy or monitoring procedures do not need the approval of an ethical committee. Additionally, the need for informed signed consent was waived. The procedure was declared to the National Institute for Health Data, with registration no. F20201023145322.

### PET acquisition and reconstruction parameters

Patients fasted for 6 h before undergoing the examination. After a 15-min rest in a warm room, they were injected intravenously with 4.0 Mb/kg of ^18^FDG. Height, weight, injected doses, capillary glycaemia at the injection time and the delay between injection and the start of the acquisition were recorded for each patient. All images were acquired and reconstructed according to the European Association of Nuclear Medicine (EANM) guidelines version 2.0^35^. PET imaging studies were performed on two different PET/CT systems:

- A PET/CT Biograph TrueV PET system (Siemens Healthineers) with 3 iterations 21 subsets with point spread function (PSF) reconstruction resulted in voxels of 2.0 × 4.0 × 4.0 mm. PET emission acquisition was performed from the skull to mid-thighs with 2 min 40 s and 3 min 40 s per bed position for normal-weight and overweight patients, respectively.
- A Vereos PET system (Philips) with 2 iterations 10 subsets with point spread function (PSF) reconstruction resulted in isotropic voxels of 2 mm^3^. PET emission acquisition was performed from the skull to mid-thighs with 2 min per bed position.

### Extraction of PET bone textural features

All images were analysed by the same reviewer with 5 years of experience in PET interpretation using MIM (MIM Software, Cleveland, OH, USA, version 5.6.5). For visual PET/CT assessment, examinations were considered to be positive in cases of one or several obvious bone focal uptakes on PET images with or without bone lesions on CT images. Doubtful diffuse and/or heterogeneous skeletal uptake was not considered a positive finding. In case of discrepancy, the examination was conjointly reviewed to reach a consensus with a second experienced nuclear medicine physician having more than 10 years of experience in PET.

For textural analysis, the skeleton volumes of interest (VOIs) from the C3 vertebra to the upper third of femurs were automatically extracted from CT images for each examination (Supplemental Fig. 1).

In the case of hip prostheses, the zone was excluded to avoid PET attenuation correction artefacts. The final CT VOIs were then transferred to PET images. All possible lymph node areas of increased FDG uptake in the vicinity of the skeleton (especially in the retroperitoneum) that could affect texture features because of a partial volume effect were checked^36^. Finally, the VOIs were saved in DICOM-RT structure format so that they could be loaded in LIFEx software version 5.1^37^. For the resampling step, 64 discrete values with a range of SUV units set to 0–30 and a spatial resampling set to 2.0 × 4.0 × 4.0 mm were used. The following PET variables were extracted:

- five conventional PET parameters: SUVmax, SUVpeak, SUVskewness, SUVkurtosis and SUVexcessKurtosis
- six grey-level co-occurrence matrix (GLCM) parameters: inverse difference, angular second moment, variance, correlation, joint entropy and dissimilarity
- three neighbourhood grey-level different matrix (NGLDM) parameters: coarseness, contrast and busyness
- eleven third-order metrics calculated from size-zone matrices: SZE (Short-Zone Emphasis), LZE (Long-Zone Emphasis), LGZE (Low Grey-Level Zone Emphasis), HGZE (High Grey-Level Zone Emphasis), SZLGE (Short-Zone Low Grey-Level Emphasis), SZHGE (Short-Zone High Grey-Level Emphasis), LZLGE (Long-Zone Low Grey-Level Emphasis), LZHGE (Long-Zone High Grey-Level Emphasis), GLNU_Z_ (Grey-Level Non-Uniformity for Zone) ZLNU (Zone Length Non-Uniformity) and ZP (Zone Percentage). Index values were calculated using a single co-occurrence matrix simultaneously considering all 13 spatial directions.

All textural features were compliant with the benchmark of the image biomarkers standardisation initiative^38^.

### Statistical analysis

Quantitative data are presented as the mean ± standard deviation (SD) or median (interquartile range) when appropriate. Characteristics of populations and PET radiomics were compared using Fischer’s exact tests for discrete variables and Mann–Whitney tests for continuous variables with Bonferroni correction. Both BMB and visual PET assessment as described above were taken as the gold standard for the patient’s classification. BMB −/PET − patients were considered disease-free patients (bone-_NEGATIVE_ patients), whereas BMB +/PET −, BMB −/PET + and BMB +/PET + patients were considered as bone-_POSITIVE_ patients. A least absolute shrinkage and selection operator (LASSO) regression algorithm with tenfold cross-validation was used to select features of interest, namely, those with nonzero coefficients. This regression method performs both variable selection and regularization to enhance the prediction accuracy and interpretability of the resulting statistical model^39^. A prediction score (pred.score) was computed for each patient by means of a linear regression combining all selected PET variables. Receiver operating characteristic curves (ROCs) were used to define the optimal pred.score cut-off value for the diagnosis of BMI by maximizing the sensitivity and specificity according to the Youden index and for diagnostic performance comparisons using the DeLong et al. methodology. Finally, Spearman correlation tests were used to determine the relationship between biological variables and PET radiomics of interest. Statistical analysis and figure conception were performed using XLSTAT software (XLSTAT 2019: Data Analysis and Statistical Solution for Microsoft Excel. Addinsoft).

### Ethical approval

The authors are accountable for all aspects of the work and guarantee that questions related to the accuracy or integrity of any part of the work are appropriately investigated and resolved. All procedures performed in the studies involving human participants were in accordance also the ethical standards of the institutional and/or national research committee and with the 1964 Helsinki Declaration (as revised in 2013) and its later amendments or comparable ethical standards.

### Consent to participate and for publication

In accordance with European regulations, French observational studies without any additional therapy or monitoring procedures do not need the approval of an ethics committee. Additionally, the need for informed signed consent was waived. Nevertheless, global information for people participating in research was provided, including a specific paragraph on the possibility of using health data for research purposes. The patient had the right to oppose the transmission of data covered by medical confidentiality that may be used and processed in the context of this research. The procedure was declared to the National Institute for Health Data with the registration no. F20201023145322.

## Supplementary Information

Supplementary Figure 1.

## Data Availability

The data supporting the conclusions of this article will be made available by the authors, upon reasonable request.
