# Supplementary figures and images for: Diagnostic value of baseline 18FDG PET/CT skeletal textural features in follicular lymphoma
Source: Sci Rep. 2021 Dec 10;11:23812. doi: 10.1038/s41598-021-03278-9 (PMC8664828; doi:10.1038/s41598-021-03278-9)

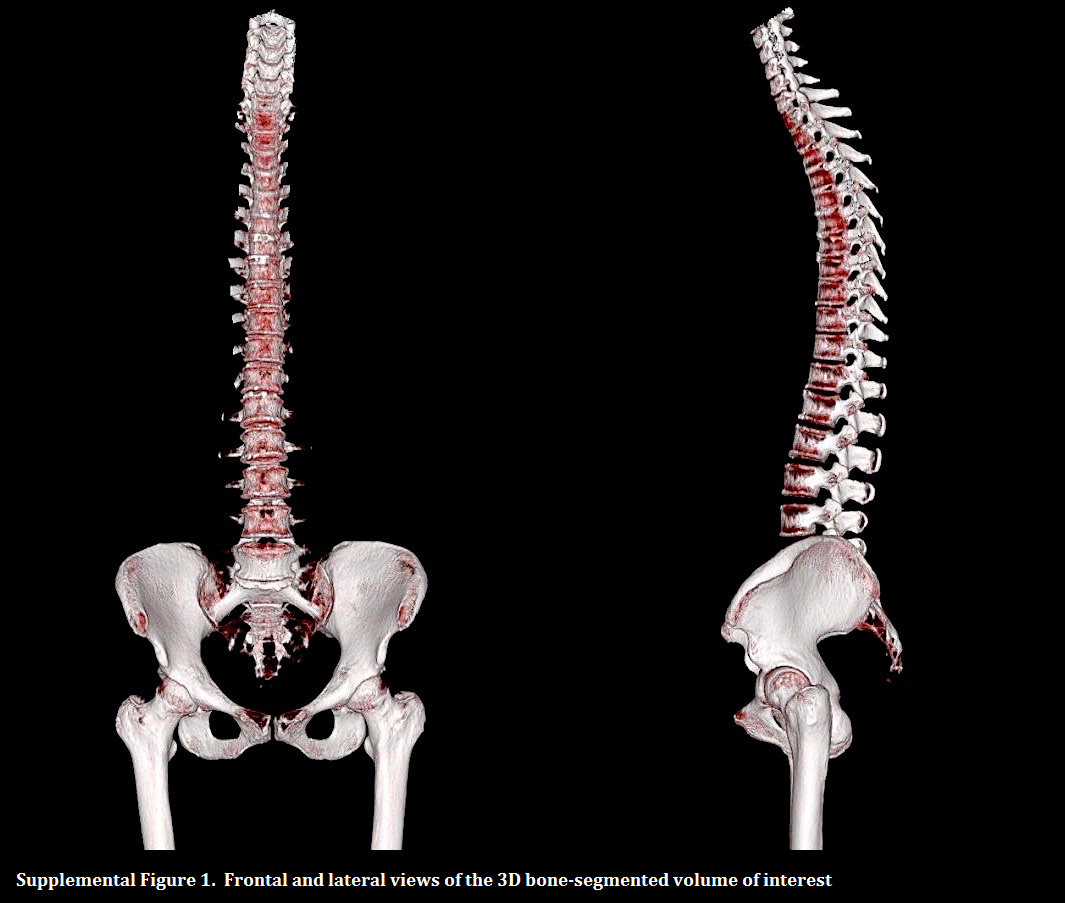

Supplement: Supplementary file 1 — Supplementary Figure 1. [file 41598_2021_3278_MOESM1_ESM.tif]
